# Trace element and temperature effects on microbial communities and links to biogas digester performance at high ammonia levels

**DOI:** 10.1186/s13068-015-0328-6

**Published:** 2015-09-22

**Authors:** Maria Westerholm, Bettina Müller, Simon Isaksson, Anna Schnürer

**Affiliations:** Department of Microbiology, Uppsala BioCenter, Swedish University of Agricultural Sciences, Box 7025, 750 07 Uppsala, Sweden

**Keywords:** Syntrophic acetate-oxidising bacteria, Acetogens, *fhs*, Methanogens, *mcrA*, VFA, Hydrogen

## Abstract

**Background:**

High levels of ammonia and the presence of sulphide have major impacts on microbial communities and are known to cause operating problems in anaerobic degradation of protein-rich material. Operating strategies that can improve process performance in such conditions have been reported. The microbiological impacts of these are not fully understood, but their determination could help identify important factors for balanced, efficient operation. This study investigated the correlations between microbial community structure, operating parameters and digester performance in high-ammonia conditions.

**Method:**

Continuous anaerobic co-digestion of household waste and albumin was carried out in laboratory-scale digesters at high ammonia concentrations (0.5–0.9 g NH_3_/L). The digesters operated for 320 days at 37 or 42 °C, with or without addition of a trace element mixture including iron (TE). Abundance and composition of syntrophic acetate-oxidising bacteria (SAOB) and of methanogenic and acetogenic communities were investigated throughout the study using 16S rRNA and functional gene-based molecular methods.

**Results:**

Syntrophic acetate oxidation dominated methane formation in all digesters, where a substantial enhancement in digester performance and influence on microbial community by addition of TE was shown dependent on temperature. At 37 °C, TE addition supported dominance and strain richness of *Methanoculleus bourgensis* and altered the acetogenic community, whereas the same supplementation at 42 °C had a low impact on microbial community structure. Both with and without TE addition operation at 42 °C instead of 37 °C had low impact on digester performance, but considerably restricted acetogenic and methanogenic community structure, evenness and richness. The abundance of known SAOB was higher in digesters without TE addition and in digesters operating at 42 °C. No synergistic effect on digester performance or microbial community structure was observed on combining increased temperature with TE addition.

**Conclusions:**

Our identification of prominent populations related to enhanced performance within methanogenic (high dominance and richness of *M. bourgensis*) and acetogenic communities are valuable for continued research and engineering to improve methane production in high-ammonia conditions. We also show that a temperature increase of only 5 °C within the mesophilic range results in an extreme dominance of one or a few species within these communities, independent of TE addition. Furthermore, functional stable operation was possible despite low microbial temporal dynamics, evenness and richness at the higher temperature.

**Electronic supplementary material:**

The online version of this article (doi:10.1186/s13068-015-0328-6) contains supplementary material, which is available to authorized users.

## Background

Decreased emissions of greenhouse gases and a lower human impact on climate change are strong incentives for replacing fossil fuel with biogas for generation of heat and electricity and vehicle fuel. Because of the high methane yield potential and high content of plant-available ammonia in the digestion residues, commercial biogas plants are keen to use protein-rich materials, such as animal manure, thin stillage, fish processing residues and slaughterhouse waste, as feedstock [1–3]. Unfortunately, anaerobic degradation of proteinaceous materials is often associated with digester instability, as indicated by reduced methane production rate, fluctuations in pH and alkalinity, and high effluent concentrations of volatile fatty acids (VFA), such as acetate and propionate [4]. Direct microbial toxicity effects of ammonia and/or the release of sulphide are often cited as reasons for process disturbance [5–7]. Sulphide is not only toxic to various microbial populations, but also forms complexes with metals, which can decrease the bioavailability of trace elements essential for microbial activity [6, 8].

The inhibitory effects of ammonia and trace element deficiency are considered to be most pronounced in latter parts of the degradation process, which involve the activity of hydrogen/formate-utilising (hydrogenotrophic) or acetate-utilising (aceticlastic) methanogens. This in turn influences reaction pathways higher up in the degradation chain [9]. As regards ammonia, methanogen sensitivity is commonly most distinctive within the aceticlastic community [10–14]. Acclimatisation of the microbial community at high ammonia levels (>3.0–3.3 g NH_4_^+^-N/L; 0.14–0.28 g NH_3_/L at 37 °C) has been shown to involve development of syntrophic acetate oxidation (SAO) coupled with hydrogenotrophic methanogenesis as the dominant pathway for acetate conversion [15–20]. The initial reaction in this two-step reaction is performed by a group of bacteria often referred to as syntrophic acetate-oxidising bacteria (SAOB). Genetic and enzymatic studies have indicated that several species with known syntrophic acetate-oxidising capability use the Wood–Ljungdahl pathway, both in an oxidative and a reductive way, and are therefore assigned to the acetogenic bacteria [21–24].

Syntrophic acetate-oxidising microorganisms differ both biochemically and in growth characteristics to aceticlastic methanogens [22, 25, 26]. This could be important when considering process design or optimisation of biogas plant operation, which is conventionally designed to support the aceticlastic microorganisms. Consequently, the performance of a process operating under conditions known to restrain aceticlastic methanogens could potentially be improved by exploiting the influence of SAOB. In addition, consideration of syntrophic propionate-oxidising bacteria (SPOB) might also be of importance as non-reversible accumulation of propionate typically appears as frequent concern during operation at high ammonia [27]. Recent studies of high-ammonia digesters dominated, or indicatively dominated, by SAO have identified two operation management strategies with positive impacts on digester performance. These are increasing the temperature (from 37 to 44 °C) [28] and adding trace elements [27, 29]. Laboratory experiments on defined syntrophic acetate-oxidising cultures have also shown optimised methane production rates at around 42–44 °C [26], further indicating potential for enhanced digester performance by operating the SAO-mediated process within this temperature range. The addition of trace elements is suggested to meet microbial requirements in metabolic pathways associated with methanogenesis [27, 29], since elements such as iron (Fe), nickel (Ni), cobalt (Co) and tungsten (W) are essential for methanogenic enzymes and cofactors [30, 31]. Furthermore, the nickel-containing carbon monoxide dehydrogenase (CODH) is a key enzyme in the Wood–Ljungdahl pathway, which is used by both methanogens and acetogens [22, 32] and known to be active in syntrophic co-cultures [21, 23]. Thus, the improved productivity and the lower acetate and propionate levels of the SAO-dominated process in response to trace element supplementation and increased operating temperature to 42–44 °C is most likely attributable to a more efficient microbial community. However, to fully understand the links between microbial composition and digester performance and adjustments in operating temperature and addition of trace elements further research is required. The objective of this study was therefore to comprehensively investigate the impact of these two operating parameters on microbial community composition and process performance. Their impacts were assessed in a long-term study of high-ammonia digesters dominated by SAO processes. 16S rRNA and functional gene-based molecular analyses were used to determine the methanogenic, acetogenic, syntrophic acetate-oxidising and syntrophic propionate-oxidising community structures, with the intention of identifying indicators of the presence of key microorganisms related to enhanced productivity.

## Results

### Digester performance and the methanogenic pathway

The obtained ratios of ^14^C-labelled carbon dioxide to methane (^14^CO_2_/^14^CH_4_) in tracer analyses confirmed SAO as the dominant pathway for methane formation from acetate in all four digesters and the pH was relatively stable in the digesters throughout operation (Table 1). The obtained levels of ammonium-nitrogen (5.4–5.8 g NH_4_^+^-N/L) were in accordance to previous study, where similar conditions for digester operation were applied [16]. Owing to the higher operating temperature, the ammonia (NH_3_) concentration was slightly higher in D42 and D^TE^42 than in the 37 °C digesters (Table 1). The hydrogen sulphide (H_2_S) level in the produced gas was considerable higher in D37 and D42 (>5000 ppm) compared to the digesters supplemented with a trace element mixture including iron (TE), which had a concentration between 200 and 500 ppm. Analyses of metal concentration showed higher levels of Fe, Co and Ni in digesters D^TE^37 and D^TE^42, whereas there was no difference in selenium (Se) content between any of the digesters. Molybdenum (Mo) was higher in the TE-supplemented digesters at the sampling point in period 1 but, similarly to Se, below the detection limit in period 3 (Additional file 1: Table S1). Methane yield and VFA concentrations in digesters D37, D42 and D^TE^37 were relatively consistent throughout the course of the trial. However, in digester D^TE^42 specific methane production and propionate concentration gradually altered over time. In order to clearly illustrate these changes, digester performance parameters are presented in Table 2 as average values within periods 1, 2 and 3, comprising 0–110, 111–220 and 221–320 days of operation, respectively.

**Table 1:** Process parameters and operating conditions in the digesters operating with and without trace element addition at 37 and 42 °C

Digester	Operating parameters		NH_4_^+^-N (g/L)	NH_3_ (g/L)	^14^CO_2_/^14^CH_4_ ratio	Average pH
	Temperature (°C)	Trace element addition				
D37	37	–	5.8	0.4–0.7	9.1 ± 2.4	7.9 ± 0.1
D42	42	–	5.6	0.6–1.5	20 ± 1.8	8.1 ± 0.2
D^TE^37	37	+	5.4	0.6–0.9	21 ± 5.1	8.1 ± 0.1
D^TE^42	42	+	5.4	0.8–1.2	14 ± 1.7	8.1 ± 0.1

pH was measured weekly and ammonium-nitrogen (NH_4_^+^-N) was measured during period 2

**Table 2:** Average performance parameters of the digesters during operational periods 1, 2 and 3 (days 0–110, 111–220 and 221–320, respectively)

Digester	Period	Spec. methane production (N L/gVS day)	Av. methane content of the gas (%)	Degree of degradation (%)^a^	VFA (g/L)^b^			
					Prop	Ace	Val	But
D37	1	0.22 ± 0.02	57 ± 2	69	5.6 ± 1.1	2.8 ± 0.6	2.6 ± 0.3	0.4 ± 0.2
	2	0.25 ± 0.02	62 ± 2	68	3.4 ± 0.8	3.4 ± 0.3	2.8 ± 0.6	0.9 ± 0.6
	3	0.23 ± 0.01	60 ± 2	72	4.2 ± 0.8	2.9 ± 0.5	2.2 ± 0.4	0.3 ± 0.1
D42	1	0.23 ± 0.02	57 ± 3	69	6.3 ± 0.7	1.5 ± 0.5	1.7 ± 0.8	–
	2	0.24 ± 0.02	61 ± 3	66	6.1 ± 2.0	2.3 ± 0.7	2.0 ± 0.6	–
	3	0.25 ± 0.01	60 ± 1	73	6.2 ± 2.0	1.7 ± 0.3	1.8 ± 0.5	–
D^TE^37	1	0.31 ± 0.02	59 ± 2	84	1.0 ± 1.8	–	–	–
	2	0.33 ± 0.02	61 ± 1	82	–	–	–	–
	3	0.33 ± 0.03	62 ± 2	89	–	–	–	–
D^TE^42	1	0.26 ± 0.02	59 ± 2	78	4.0 ± 1.3	0.2 ± 0.1	–	–
	2	0.30 ± 0.01	62 ± 1	74	0.4 ± 0.4	0.3 ± 0.3	–	–
	3	0.30 ± 0.03	61 ± 2	88	0.1 ± 0.1	0.1 ± 0.1	–	–

^a^A rough estimation method [62] was included in the calculation to compensate for VFA losses in the determination of dry matter. The standard deviations in the triplicate analyses in each period were >0.001

^b^–, below detection limit (<0.1 g/L); Prop, propionate; Ace, acetate, Val, isovalerate; But, isobutyrate

The reference digester D37 had an average methane (CH_4_) yield of around 0.22–0.25 n L/g volatile solids (VS)/day throughout the course of the study and the acetate, propionate, isobutyrate and isovalerate concentrations were high, in total 9–10 g/L (Table 2). The addition of TE mixture during operation at 37 °C increased the average methane yield in digester D^TE^37 by 35–42 % compared with D37, and the VFA levels remained low or below the detection limit in the D^TE^37 digester throughout the course of the study. Increasing the operating temperature without TE addition had no impact on methane yield and resulted in a large amount of VFA accumulation, in particular propionate, which impeded the operation of D42 (Table 2). However, TE addition at 42 °C resulted in 12, 22 and 23 % increased methane yield in relation to D42 in period 1, 2 and 3, respectively. This increase in yield over time occurred in parallel with a decrease in propionate concentration from 4 g/L to below the detection limit (>0.1 g/L) in D^TE^42 (Table 2). Comparing the TE-supplemented digesters revealed that operation at 42 °C instead of 37 °C resulted in lower methane yield (−18 %) and increased level of propionate in the initial operating period of D^TE^42. However, in line with the propionate degradation over time, a gradual increase in methane production ensued in D^TE^42, and in period 3 digester D^TE^42 had only 8 % lower methane yield than D^TE^37. Addition of TE increased the degree of degradation in both digesters, but became most prominent in digester D^TE^42 during periods 2 and 3 (Table 2).

### Kinetic study and batch degradation assays

**Fig. 1:**
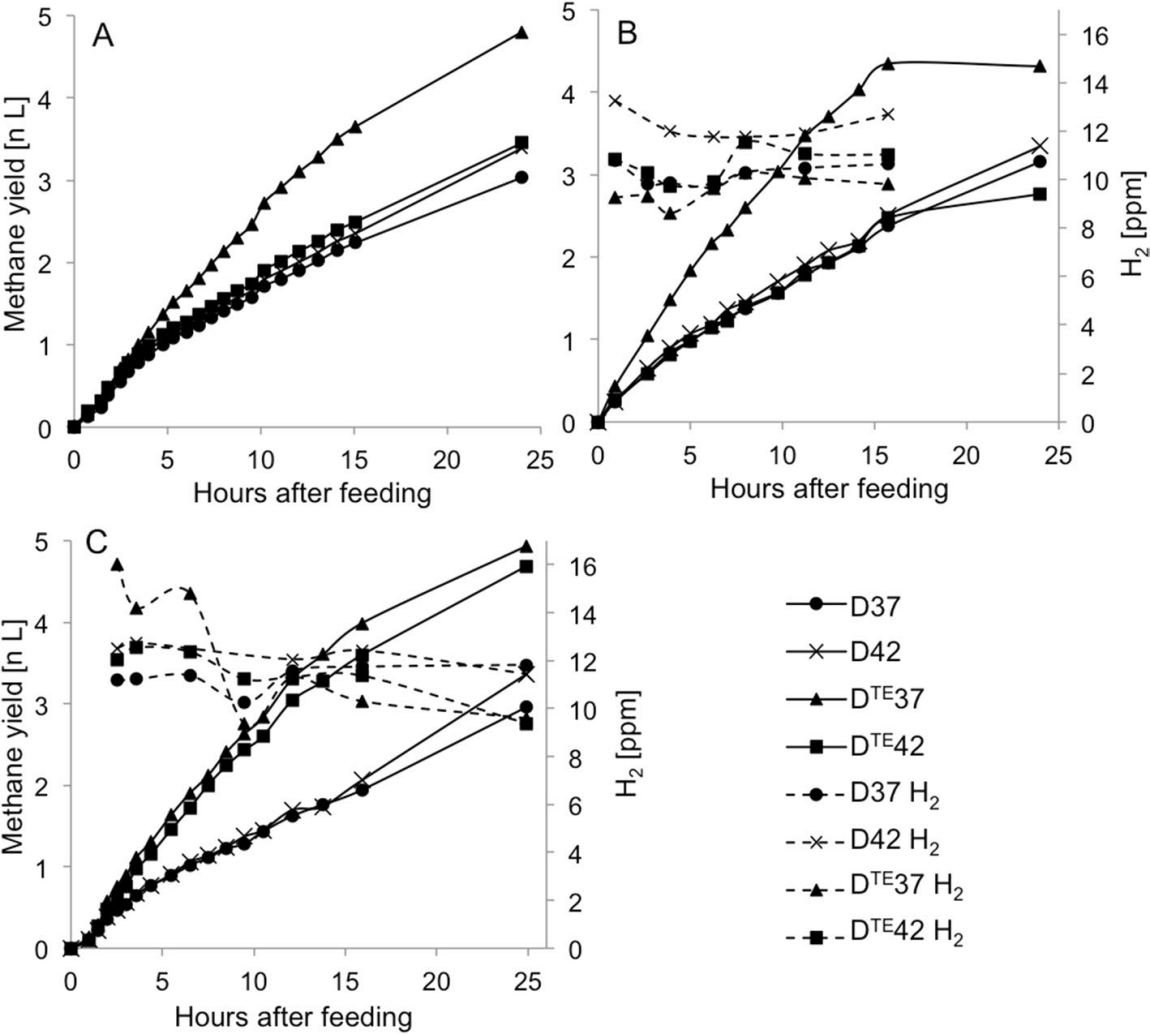
Methane production and hydrogen partial pressure after feeding of the continuous laboratory-scale digesters. The kinetics of methane production in the anaerobic digesters was estimated after **a** 20, **b** 160 and **c** 311 days of operation, in order to represent operating period 1, 2 and 3, respectively. The relative hydrogen partial pressure in the digesters was analysed on days **b** 160 and **c** 311

The kinetics of methane production after feeding were analysed on days 20, 160 and 311, in order to represent periods 1, 2 and 3, respectively. The results revealed considerably higher methane production rate in digester D^TE^37 than in the other digesters in periods 1 and 2 (Fig. 1a, b). In period 2, D^TE^37 also had the lowest partial hydrogen pressure. The results of digester D^TE^42 on day 311 differed from those obtained for the previous occasions and clearly reflected the improved performance of the digester in period 3 (Fig. 1c). Furthermore, measurement of partial hydrogen pressure in D^TE^42 before feeding and 24 h after feeding on day 311 indicated levels similar to the relatively low value in digester D^TE^37.

In the batch assay experiment, which was performed in period 2, the number of days needed for formation of 50, 80 and 100 % of total methane potential varied between the digesters. However, the rate was clearly faster when the test was started with inoculum from the digesters with TE addition (Table 3). Progress curves showing the average net concentration of acetate and propionate in the batch assays also revealed clear differences (Additional file 1: Figure S1). The acetate and propionate originating from degradation of the added substrate material diminished within 13–22 days in batches started with inoculum from D^TE^37 and D^TE^42. However, in batches where biomass was taken from digesters without supplementation, about 26 and 40 days were needed for complete degradation of VFA at 42 and 37 °C, respectively. In those cases elevated levels of isovalerate (up to 0.6–0.8 g/L) were also detected, confirming observations made in the parent digesters (Table 2).

**Table 3:** Days required for formation of 50, 80 and 100 % of the total methane potential based on results from batch trials. The digester from which the inoculum digestate was taken is shown in brackets

Methane production of total potential (%)	1 (D37)	2 (D42)	3 (D^TE^37)	4 (D^TE^42)
100	24 ± 0.1	19 ± 2	12 ± 1	14 ± 1
80	21 ± 1	17 ± 2	11 ± 2	12 ± 1
50	17 ± 1	14 ± 2	9 ± 2	10 ± 2

### Analyses of the microbial communities

#### Methanogenic abundance and community structure

**Fig. 2:**
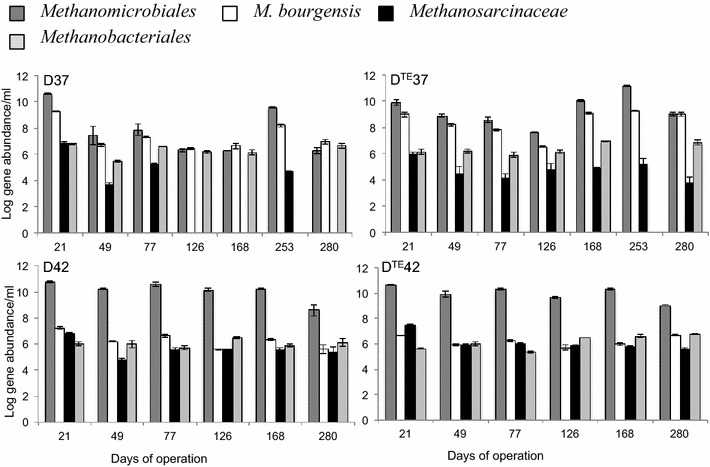
Methanogenic abundance. Logarithmic gene copies/mL of *Methanomicrobiales*, *M. bourgensis*, *Methanosarcinaceae* and *Methanobacteriales* at 21, 49, 77, 126, 168 and 280 days of operation. Additional qPCR analyses of digesters D37 and D^TE^37 were carried out for day 253, but *Methanobacteriales* was not included in these due to the persistent values of this group in the other samples. *Methanosarcinaceae* was below detection limit at day 126, 168 and 280 and *bars* for this family thus do not appear at these time points

Quantitative PCR (qPCR) results obtained with primers targeting the 16S rRNA gene of methanogens belonging to *Methanomicrobiales*, *Methanosarcinaceae*, *Methanobacteriales* and *Methanoculleusbourgensis* are shown in Fig. 2. Genes related to *Methanosaetaceae* did not appear above the detection limit in any digester. *Methanobacteriales* varied between 2 × 10^5^ and 8 × 10^6^ gene copies/mL in all sampling points and no significant difference was revealed between the digesters. *M*. *bourgensis* comprised the major part of *Methanomicrobiales* and both increased in abundance with TE addition at 37 °C. The levels of *Methanosarcina*ceae was relatively low at 37 °C independent of TE addition and detected values varied between 4 × 10^3^–6 × 10^6^ gene copies/mL. In D^TE^37 *Methanosarcina*ceae was below detection limit of 1 × 10^3^ gene copies/mL at day 126, 168 and 280 (Fig. 2). Comparisons of the digesters operating at 42 °C with and without addition of TE revealed no significant difference in gene abundance of any methanogenic group. However, the enhanced temperature in the non-supplemented digester (D42) caused a significant increase of *Methanomicrobiales* and *Methanosarcinaceae* compared to D37, whereas the level of *M*. *bourgensis* decreased. *Methanosarcinaceae* was significantly higher in D^TE^42 than in D^TE^37, whereas the difference in temperature between these digesters did not affect the levels of *Methanomicrobiales*. However, as observed for the non-supplemented digesters, *M*. *bourgensis* was lower at 42 °C than at 37 °C. Furthermore, in D42 and D^TE^42 the gene abundance of *Methanomicrobiales* and *M*. *bourgensis* appeared to be less dynamic compared to the 37 °C digesters (Fig. 2).

**Fig. 3:**
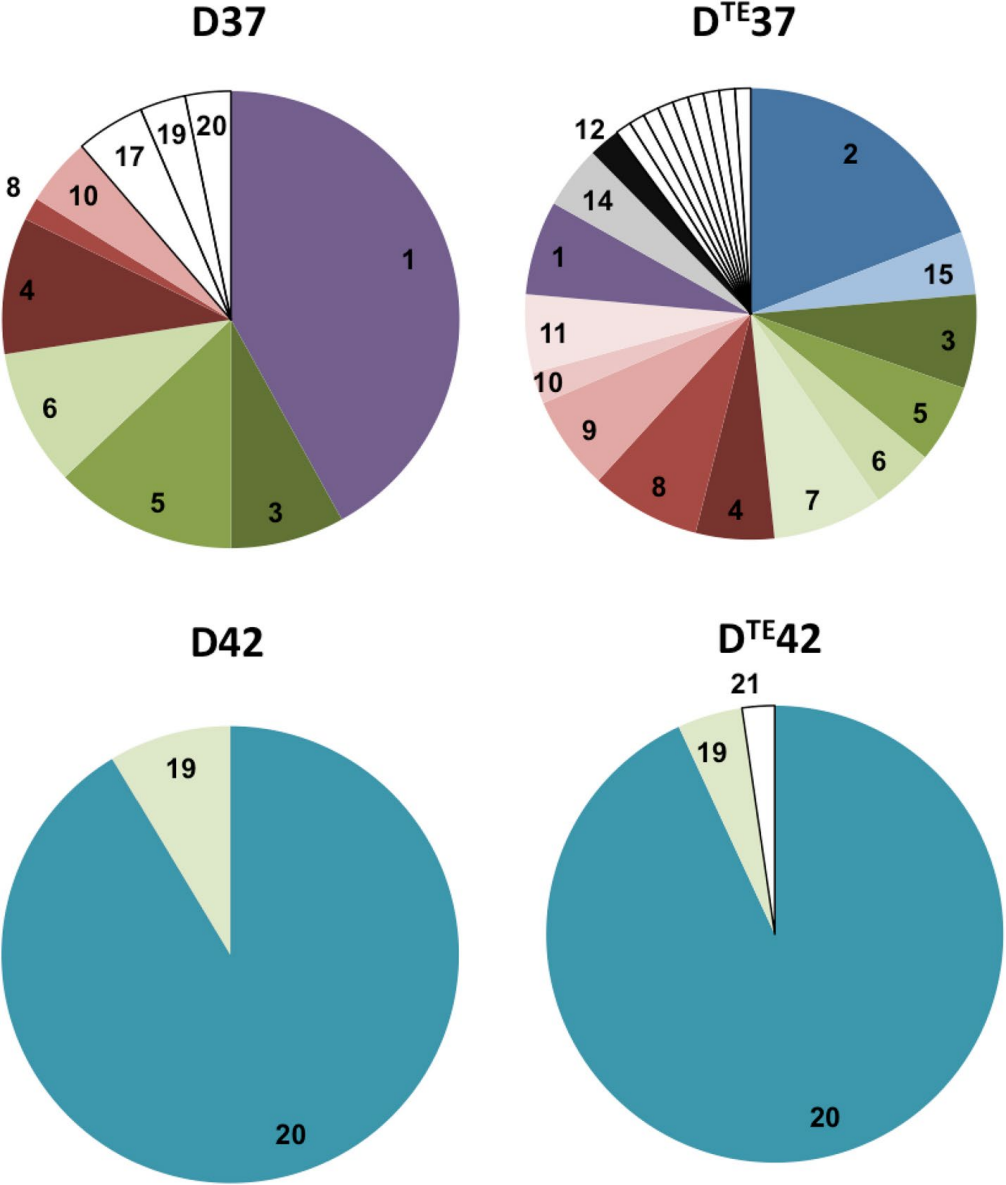
Methanogenic community structure. Relative OTU distributions of *mcrA* genes retrieved in construction of clone libraries from digesters D37, D^TE^37, D42 and D^TE^42 at day 280 (period 3) based on 99 % identity threshold. *Figures in the sectors* represent OTU numbers. OTU1 and OTU19 had low identity (<79 %) to previously available *mcrA* genes. Nucleotide/amino acid sequence identity/similarity (%) to *M. bourgensis* OTU2: 92/89; OTUs 3–9: 97–99/≥89; OTU20: 92/89. Sectors with similar colour but different tunes are OTUs assembling based on 97 % identity threshold and referred to as assemblage *A* (*blue*), *B* (*red*) and *C* (*green*). *Uncoloured* sectors are OTUs and single *mcrA* genes with relatively low abundance. Frequencies and identities of the OTUs are listed in Additional file 1: Table S1

Over 300 partial *mcrA* (methyl coenzyme-M reductase) sequences were recovered and analysed in the clone libraries. About 4 % of the total clones originated from unspecific amplification and were therefore excluded from further analysis. The recovery rate of the libraries was 74 % in D^TE^37, 83 % in D37, 97 % in D42 and 97 % in D^TE^42. The relative abundances of partial genes recovered are shown in Fig. 3, either grouped as operational taxonomic units (OTUs) or as single clones. The grouping of *mcrA*-gene sequences based on ≥99 % identity, or on ≥97 % (data not shown), provided a higher number of OTUs and higher number of separate single sequences in D^TE^37 than in D37. The profiles of the 42 °C digesters were identical irrespective of the identity cut-off value chosen (≥97 or ≥99 %).

**Fig. 4:**
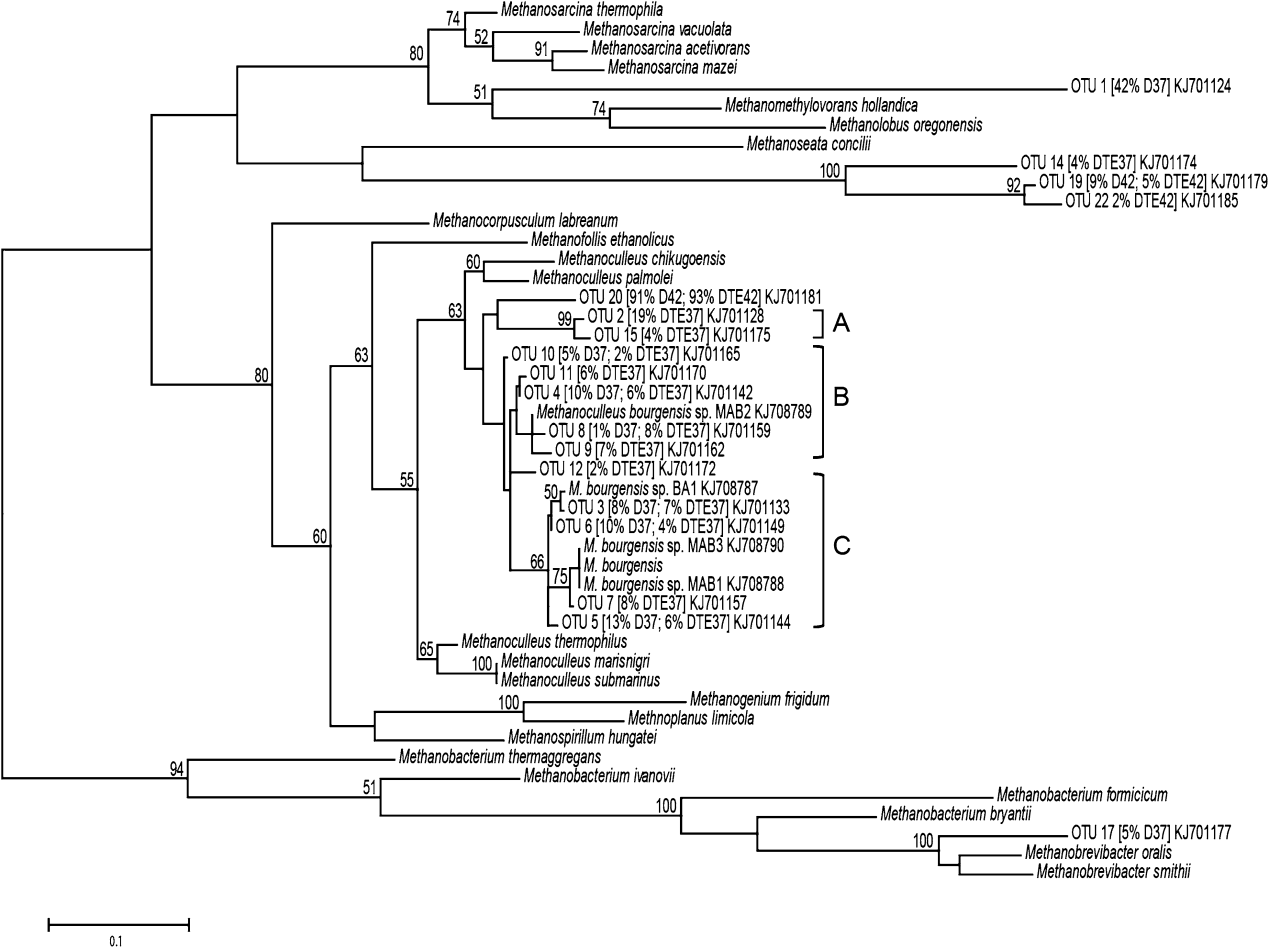
Phylogenetic relationships of reference methanogens and *mcrA* sequences retrieved from the digesters. The *mcrA* clone libraries were obtained from DNA samples collected on day 280, i.e. in period 3, from digesters D37, D^TE^37, D42 and D^TE^42. The relative abundances of the OTUs (99 % identity threshold) in the digesters are given in brackets and the GenBank accession numbers for representative sequences are specified at the end. Assemblages *A*, *B* and *C* are OTUs resembling with ≥97 % identity, and their closest relatives. The *scale bar* represents 10 % sequence divergence. *Values at the nodes* are the percentages of 1000 bootstrap replicates; values below 50 % are not shown

The position of each OTU and the accession number of reference sequences are shown in the phylogenetic tree (Fig. 4), while the closest relative(s) based on nucleotide sequence and deduced amino acid sequence identity are assigned in Additional file 1: Table S2. The brackets in Fig. 4 bundle those OTUs sharing at least ≥97 % identity and the respective closest relative. The assemblages are designated A (OTU2, 15), B (OTU4, 8–11) and C (OTU3, 5–7). OTUs in assemblage C appeared in both D37 and D^TE^37 with equal order of magnitude (25–30 % of total clones). Group B was also represented in both these digesters, but with lower relative abundance in D37 (16 % of total clones) than in D^TE^37 (28 %). Sequences included in group A were found only in D^TE^37 (24 % of total clones).

OTU1, highly dominant in D37 (41 % of the total clones), had low nucleotide sequence identity (<79 %) to previously available *mcrA* genes. However, the deduced amino acid was revealed to have 88 % similarity to *Methanosarcinaacetivorans* and *Methanosarcina mazei*. The other highly prevalent OTUs (3–6, 10 and 12) in digester D37 were closely related to *M. bourgensis* (97–99 % nucleotide identity, ≥97 % *mcrA* amino acid similarity), and together accounted for 50 % of the total clones. About 5 % of the gene sequences retrieved in D37 (OTU17) most closely resembled *Methanobrevibacter smithii* (89 % nucleotide identity, 97 % *mcrA* amino acid similarity). In D^TE^37, 19 % of the retrieved clones assembled into OTU2 (92 % nucleotide identity, 97 % amino acid similarity to *M. bourgensis*). OTU1, highly dominant in D37, also appeared in D^TE^37, but was of considerably lower relevance (7 %) than in the non-supplemented digester. In D^TE^37, the OTUs closely affiliating to *M. bourgensis* (97–99 % nucleotide identity; OTUs 3–12) comprised 64 % of the total clones, but as many as 96 % of the clones had *M. bourgensis* as closest relative (92–99 % nucleotide identity; OTUs 2–12 and 15). The closest relative to OTU14 (4 % of clones from D^TE^37) was *Methanomassiliicoccus luminyensis* (83 % nucleotide identity, 94 % similarity). OTU20 dominated the methanogenic community in both D42 and D^TE^42, comprising 91–93 % of the total clones, respectively. Based on the nucleotide sequence, this OTU was most closely related to *M. bourgensis* (93 %), whereas the deduced amino acid sequence had highest identity (97 %) to *Methanoculleus chikugoensis*. *mcrA* genes (OTU19) with low identity (<79 %) to previously available sequences and 97 % amino acid identity to *M. luminyensis* were also identified in the 42 °C digesters.

#### Acetogenic community

**Fig. 5:**
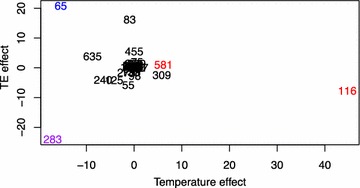
Ordination of terminal restriction fragment (T-RF) frequency of the acetogenic community (by targeting of the fhs gene) by non-metric multidimensional scaling (MDS). TE effect estimate versus temperature effect estimate for each T-RF. The T-RFs that significantly associated with TE-addition at 37 °C (65 bp), temperature without TE-addition (116 bp; 581 bp) or with the combined temperature increase and TE addition (283 bp) are coloured* blue*,* red* and* purple*, respectively

The profiles of the acetogenic communities in the digesters on days 77, 168 and 280, obtained by terminal restriction fragment length polymorphism (T-RFLP) analysis using the functional gene *fhs* encoding the enzyme formyltetrahydrofolate synthetase (FTHFS), are displayed in Additional file 1: Figure S2. Ordination of terminal restriction fragment (T-RF) frequency and abundance by using a paired *t* test illustrated that a few of the most abundant T-RFs differed from the digester core community, apparently significantly affected by TE addition (*x* axis) and/or increased temperature (*y* axis) (Fig. 5). T-RF 65 bp positively correlated with TE addition at 37 °C (T-RFLP gene frequencies between 10 and 31 % in D37; 33–44 % in D^TE^37), whereas at this temperature TE addition had a negative impact on the abundance of T-RF 283 bp (22–33 % in D37; 3 % in D^TE^37). TR-F 283 bp was also negatively affected by operation at 42 °C instead of 37 °C, both with and without TE addition (22–33 % in D37; 6–17 % in D42; >17 % in D^TE^42). In contrast, temperature had a strong positive influence on T-RF 116 bp (>16 % in D37; 33–72 % D^TE^42) and a moderate positive influence on the abundance of T-RF 581 bp (>3 % at 37 °C and >8 % at 42 °C).

The majority of the most abundant T-RFs were assigned to partial *fhs* sequences recovered in the clone libraries of D^TE^37 and D^TE^42 at day 168. The relative OTU distribution is displayed in Additional file 1: Figure S3 and the accession numbers and the assignments of retrieved *fhs* sequences are listed in Additional file 1: Table S3. In total, 23 OTUs and genotypes were recovered from D^TE^37, which was twice as much as from D^TE^42. Of these, only OTU_*fhs*_32, 29, 30, 26 and the genotype KJ_701058 were recovered from both temperature conditions (Additional file 1: Table S3, Figure S3). OTU_*fhs*_31, 32, 33, 21 and 25 lacked the restriction site for *Hpy*188III and are thereby all represented by T-RF 635 bp in Additional file 1: Figure S2. Consequently, temporal dynamics or differences between digesters as regards these OTUs were not tracked by T-RFLP. However, the *fhs* clone library disclosed higher frequency of OTU_*fhs*_32 in D^TE^37 than in D^TE^42, whereas OTU_*fhs*_31, 33, 21 and 25 were only retrieved from D^TE^37.

**Fig. 6:**
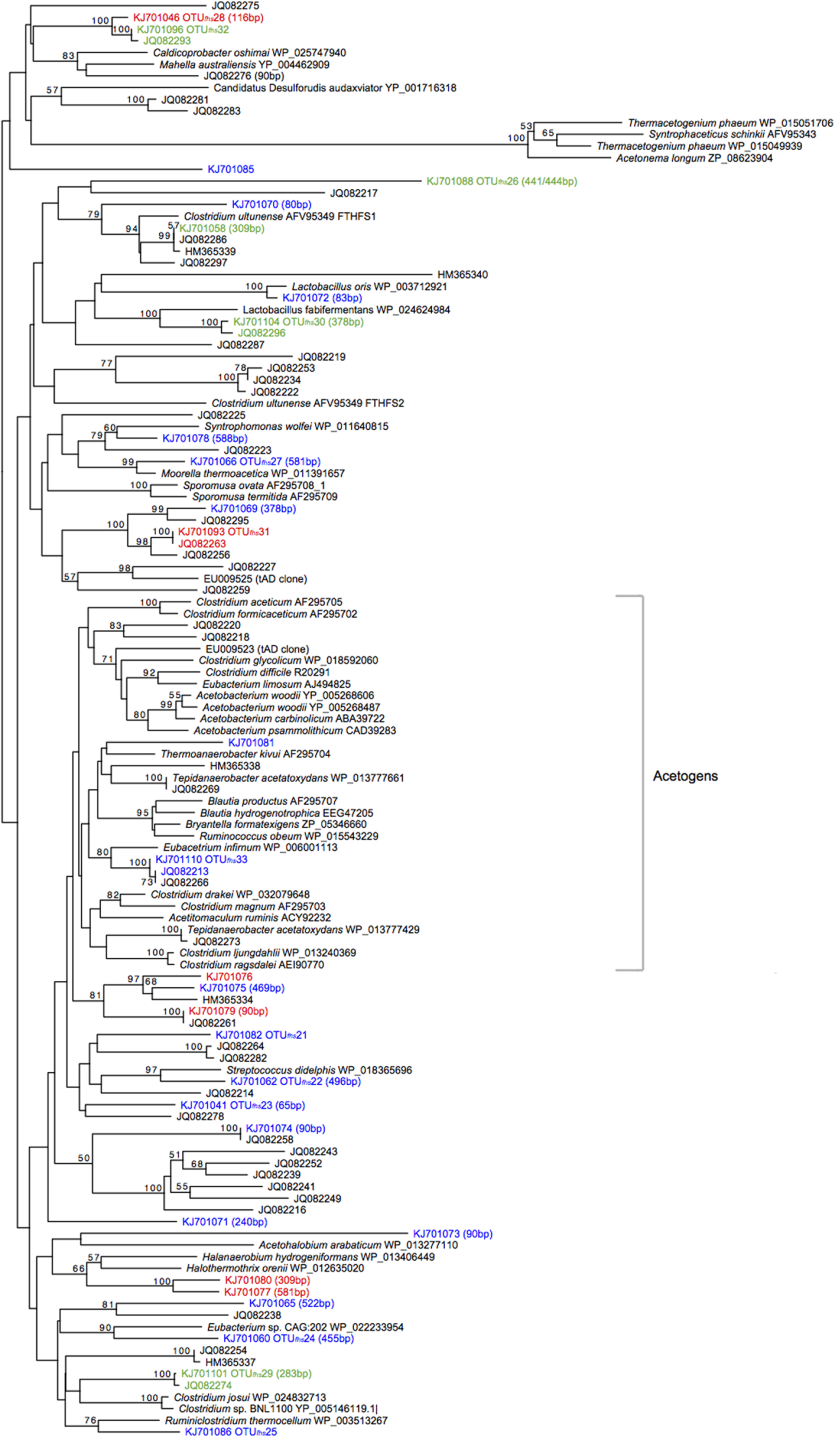
Phylogenetic placements of deduced FTHFS amino acid sequences of the partial *fhs* sequences retrieved from D^TE^37 and D^TE^42. The *fhs* clone libraries were constructed of DNA samples collected on day 280, i.e. in period 3, from the TE-supplemented digesters. Reference acetogens are shown in *bracket*. The *fhs* sequences retrieved from D^TE^37, D^TE^42, or both, are coloured *blue*, *red*, or *green*, respectively. The terminal restriction fragment sizes are given in parentheses and the GenBank accession Nos. for representative sequences are specified at the end. The *scale bar* represents 20 % sequence divergence. *Values at the nodes* are the percentages of 1000 bootstrap replicates; values below 50 % are not shown

The recovered *fhs* genes distinguished phenotypically from each other and had partly low identities to known acetogens (Fig. 6). However, the phylogenetic tree constructed from deduced FTHFS amino acid sequences illustrated similarity with partial *fhs* sequences recovered from another previously investigated mesophilic AD process operating with high ammonia levels [16, 33] (Genbank Popset JQ082255-97, SAO3 clones; submitted by B. Müller and A. Schnürer). One partial *fhs* sequences retrieved from D^TE^37 (KJ_701058 T-RF 309 bp) positioned together with the FTHFS1 of *Clostridium ultunense* and was to 99 % identical to one of the previously identified *fhs* clones (JQ082286, HM365339). None of the sequences grouped together with *Syntrophaceticus schinkii* or *Thermacetogenium phaeum*, or seemed to have close relatedness to FTHFS1 and FTHFS2 of *Tepidanaerobacter acetatoxydans*. OTU_*fhs*_28 (T-RF 116), highly abundant at 42 °C, and OTU_*fhs*_32, present in both D^TE^37 and D^TE^42, were in the tree closely related to each other, and the latter was identical to *fhs* clone JQ082293. Also OTU_*fhs*_30 retrieved from both D^TE^37 and D^TE^42 and OTU_*fhs*_31 highly presented in D^TE^42 showed 99 % sequence identities to *fhs* clone JQ082296 and *fhs* clone JQ082263, respectively.

The T-RFs 65, 283 and 581 bp, which were significantly affected by temperature or/and TE addition, likely represent restriction fragments of OTU_*fhs*_23, OTU_*fhs*_29 and OTU_*fhs*_27 (Figs. 5, 6 and Additional file 1: Table S3). T-RF 283 bp (OTU_*fhs*_29), negatively correlating with TE addition and increased temperature shared 99 % identity to *fhs* clone JQ082274. T-RF 65 bp (OTU_*fhs*_23), only recovered from D^TE^37 and positively affected by TE, was also branching together with one of the previously recovered *fhs* clones (JQ082278). T-RF 581 bp positively correlating with temperature can either affiliate to OTU_*fhs*_27 or to genotype KJ_701077. Since OTU_*fhs*_27 was exclusively recovered from D^TE^37, KJ_701077, which was only recovered from D^TE^42, might rather correspond to T-RF 581 bp. The positioning in the tree revealed the thermophilic acetogen *Moorella thermoacetica* as a close relative to OTU_*fhs*_27, whereas the genotype KJ_701077 was grouped in the same branch as two members of the halotolerant *Haloanaerobiales*.

#### Abundance of total bacteria and syntrophic acetate-oxidising bacteria (SAOB)

Quantitative determination of total bacterial abundance and of the SAOB *C. ultunense*, *S. schinkii* and *T. acetatoxydans* revealed relatively consistent levels throughout the course of the study, and are therefore given as average values of all sampling points in each digester in Table 4.

**Table 4:** Average gene copy numbers per mL digester sludge of total bacteria and three syntrophic acetate-oxidizing bacteria species

Digester	Total bacteria	*S. schinkii*	*T. acetatoxydans*	*C. ultunense*
D37	4.6 ± 4.1 × 10^9^	3.0 ± 3.2 × 10^8^	2.1 ± 3.1 × 10^7^	8.2 ± 8.0 × 10^6^
D42	8.8 ± 5.6 × 10^9^	10.0 ± 7.0 × 10^8^	3.0 ± 2.3 × 10^8^	5.5 ± 2.8 × 10^7^
D^TE^37	6.8 ± 6.2 × 10^9^	7.7 ± 6.2 × 10^6^	0.9 ± 1.0 × 10^7^	6.1 ± 3.4 × 10^6^
D^TE^42	4.5 ± 3.8 × 10^9^	6.1 ± 1.7 × 10^7^	6.2 ± 4.6 × 10^7^	5.4 ± 2.4 × 10^7^

qPCR analyses were performed in triplicate DNA extractions from samples withdrawn on days 21, 49, 77, 126, 168, 253 and 280 of operation

Comparisons of the abundance of *C. ultunense* in digesters with and without addition of TE revealed no significant difference during operation at 37 or 42 °C. However, *S. schinkii* abundance was significantly (*P* < 0.05) lower in D^TE^37 and D^TE^42 than in D37 and D42, respectively. *T. acetatoxydans* appeared at lower levels in D^TE^42 than in D42, whereas the qPCR data indicated no substantial disparity between D37 and D^TE^37.

Comparisons of digesters operating at 37 °C and at 42 °C revealed significantly (*P* < 0.05) higher levels of *C. ultunense*, *S. schinkii* and *T. acetatoxydans* at the higher temperature, in digesters with and without addition of TE. No products were generated in end-point PCR analyses of digester sludge with primers targeting *T. phaeum* and *Thermotoga lettingae.*

#### Presence of syntrophic propionate-oxidising bacteria (SPOB)

PCR analyses of SPOB were conducted with primers targeting the propionate CoA-transferase gene (*pct*). No specific products were generated from digester sludge in PCR with the general pct primer or with the specific primers (pct-c1 to c7). In PCR with general pct primers and pure culture DNA of *Syntrophobacterfumaroxidans*, *Syntrophobacter sulfatireducens*, *Syntrophobacter wolinii* and *Pelotomaculum propionicicum*, products of different lengths were generated. Altered PCR conditions (decreased annealing temperature from 62 to 61 °C; exclusion of bovine serum albumin, BSA) enhanced the retrieved amount of amplicons of predicted length, but gel purification and sequencing showed that this was not a specific product. In PCR using the primer pair pct-c1 or pct-c6, designed to target *S. fumaroxidans* MPOB and *Tepidanaerobacter* sp.-related *pct*, respectively, no products were formed from genomic DNA of *S. fumaroxidans* and *T. acetatoxydans*. In PCR with the 16S rRNA targeting primer pair Synt737-f/906-r, amplicons of the correct size were formed from pure culture DNA of *S.fumaroxidans, S. sulfatireducens* and *S. wolinii*, but not from *Syntrophobacter pfennigii*. However, with these primers no products were generated in PCR analyses of the digester samples. Inhibition of PCR could be excluded by spiking experiments using pure culture DNA. The specificity of Smi103-f/442-r could not be practically assessed, due to lack of growth of *Smithellapropionica* (DSM 16934, obtained from DSMZ) and absence of product formation with this primer pair in digester samples.

## Discussion

The finding that SAO was the main mechanism for acetate degradation in all four digesters confirms previous reports of selection for the syntrophic pathway at ammonium concentrations above a threshold value of 0.14–0.28 g NH_3_-N/L at mesophilic conditions [16, 17, 19, 20, 29, 34]. In present study the use of sludge from an ammonia-adapted process, dominated by SAO and high abundance of SAOB, as inoculum [16, 35] could have influenced on the appearance of syntrophic oxidation as main pathway. However, the number of studies mentioned above, in which tracer analyses clearly confirmed syntrophic acetate oxidation at high ammonia, strongly suggests that this pathway would have been the main route for acetate degradation and enrichment of SAOB would have appeared even if an un-adapted inoculum had been used here. In contradiction to previous studies, dominance of SAO despite prevalence of a wide range of acetate concentrations (>0.1–70 mM) was demonstrated in the present study. The results thus show that it is possible to operate an SAO-dominated process at low acetate concentrations, indicating that in continuous digesters operating in high-ammonia conditions at mesophilic temperature, high acetate is not necessarily a determining factor for development of SAO. In the present study, ammonia was a factor with a strong impact and certain operating parameters (e.g. addition of TE) influenced the prevailing acetate concentration, possibly due to increased microbial activity or growth of a more proficient community.

### Impact of trace element addition

#### Digester performance, production kinetics and batch degradation assays

The disparity in performance of the four digesters studies reflected the positive impact on methane yield of addition of TE, as reported in other studies investigating anaerobic degradation of food industry and household waste [27, 29, 36, 37]. Since iron was included in the TE mixture used in this study this could also have contributed to the enhanced performance observed in supplemented digesters. Iron constrains the sulphide precipitation of available trace metals in sludge, due to the primary removal of released sulphide by the iron [2, 29, 38]. The increase in methane yield (30–37 %) achieved by TE addition would substantially increase the profits in a commercial biogas plant, although the cost of the TE mixture would need to be included. Furthermore, the methane production rate was higher already in the early part of the feeding cycle in digester D^TE^37 (all periods) and in D^TE^42 (period 3), which implies potential to increase the organic loading rate (OLR) with maintained efficiency of methane production.

Another finding supporting previous studies [27, 29, 36, 37] was that addition of TE proved to be a potent intervention against VFA accumulation, both in the semi-continuous digesters and in the batch assays. Elevated VFA levels are highly undesirable, since they represent significant loss of biogas. This was indicated by the lower methane yield in digesters D37 and D42 with their high VFA concentrations compared with D^TE^37, which operated with VFA levels below the detection limit. A probable cause of the high VFA in D37 and D42 was lack of trace elements required for enzymes involved in the removal of hydrogen, since that affects both acetate and propionate oxidation by product-induced feedback inhibition [39]. Co and Se are required for synthesis of the enzymes needed for hydrogenotrophic methanogenesis [40, 41] and deficiency of these elements has previously been reported to induce propionate accumulation in a high-ammonia mesophilic process indicatively dominated by SAO [27]. Other studies suggest a prominent role of Ni and Fe in enzyme activity in the reduction of carbon dioxide with hydrogen to methane [42]. Furthermore, availability of these nutrients could also be critical for SAOB, thereby making them necessary for efficient SAO-mediated degradation.

Interestingly, despite the slow reduction in propionate in the batch assay without TE addition, acetate remained at relatively low levels, indicating the presence of efficient acetate-degrading communities. Thus, it seems likely that the accumulation of propionate was a consequence of a less efficient propionate-degrading microbial community in the unsupplemented digesters compared with the TE digesters. However, the picture becomes more complex when considering that low concentrations of hydrogen and acetate are also known to shift several of the primary fermentation pathways towards the formation of hydrogen, carbon dioxide and acetate, which results in lower production of side products such as propionate [43]. Consequently, the low VFA level in the TE-supplemented digesters might be a combined effect of reduced formation of VFA and enhanced conversion efficiency.

Elevated VFA levels have been suggested to indicate process disturbance [44]. However, despite the high VFA levels in D37 and D42, relatively stable operation was maintained throughout the 320-day study. In this context, it should be borne in mind that the consistent operation maintained in the experimental digesters could have increased their functionality. The larger variations in input material and load typically seen at large-scale production plants would most likely compromise the functional stability observed in the experimental digesters.

#### Abundance and composition of methanogens, acetogens and SAOB

Addition of trace elements significantly increased the abundance of the hydrogenotrophic *Methanomicrobiales*, specifically *M. bourgensis*, at 37 °C, whereas it had no effect on the level of *Methanosarcinaceae*. This contradicts previous findings that addition of TE increases the abundance of *Methanosarcinaceae*, allowing it to outcompete members of *Methanomicrobiales* and *Methanobacteriales* [29]. That study defined SAO as the dominant pathway for methanogenesis of acetate, which led the authors to suggest involvement of *Methanosarcinaceae* as a hydrogenotrophic partner in SAO. Differences in operating conditions, in particular the lower ammonia concentration (0.4 g NH_3_/L) in that study compared with the present investigation, is a likely reason for the different effect of TE addition on *Methanosarcinaceae*.

The *mcrA* gene clone libraries confirmed the qPCR results by demonstrating that 50 and 64–96 % (based on >97–92 % nucleotide identity) of total recovered genes affiliated to *M. bourgensis* in D37 and D^TE^37, respectively. Methanogenic assemblages obtained by grouping based on ≥97 % sequence identity (shown in brackets in Fig. 4) visualised further structural differences between the 37 °C digesters. The higher relative abundance of strains represented by assembly B and C in D^TE^37 could be the cause, or the effect, of the improved efficiency and the low hydrogen partial pressure of this specific digester. Furthermore, several of the *mcrA* genes, in particular from D^TE^37, related to *M. bourgensis* strains MAB1, MAB2, MAB3 and BA1 [20], which have been identified as important hydrogen-utilising microorganisms in association with SAO, possibly owing to tolerance to elevated levels of ammonia and a high affinity for hydrogen [17]. This would contribute to lower hydrogen partial pressure in the system, in particular that prevailing in D^TE^37, which would positively influence the upstream fermenting microbes and subsequently the complete process.

The relatively lower abundance of *S. schinkii* in both digesters with TE addition and of *T. acetatoxydans* in D^TE^42 compared with the other digesters was interesting, particularly since this occurred concurrently with persistent dominance of the SAO pathway and enhanced performance of these digesters. *Fhs* gene-based analyses were carried out in order to investigate the presence and impact of operating parameters on bacteria with the potential to be significant contributors to the SAO process in the digesters. Statistical analysis revealed that a few T-RFs were significantly influenced by addition of TE. TE addition might have increased the competiveness of members of the SAOB community and led to reduced abundance of a particular member, as observed for *S. schinkii* and T-RF 286 bp when comparing D37 and D^TE^37. In contrast, T-RF 65 bp seemed to have a competitive advantage. At higher temperature, the effect of TE addition on the community dynamics appeared clearly reduced, most likely due to co-occurrence of loss of species richness. Another impact factor to consider might be the prevailing VFA levels. The T-RFLP profile indicated a link between VFA and T-RF 283 bp. This fragment was relatively abundant in D37 and D42 (which had high VFA levels) and also appeared in D^TE^42 at first, but then gradually decreased in abundance concurrently with the decline in VFA in this digester (Additional file 1: Figure S3).

### Impact of temperature

#### Digester performance, production kinetics and batch degradation assays

Operation at 42 °C, instead of the commercially more common range 35–38 °C, had a low (D42) or transient negative (D^TE^42, periods 1–2) impact on methane yield. Similarly, in previous studies on the processing of energy crops, temperature fluctuations within correlating ranges had a low impact on methane yield [45], whereas increasing the temperature from 38 to 44 °C considerably increased the methane yield from nitrogen-rich distiller’s waste [28]. An important aspect when considering the impact of operating temperature on digester performance is the effect on the ammonia level. Increased temperature shifts the equilibrium between NH_4_^+^ and NH_3_ towards the latter, which is mainly responsible for inhibition of the microbial community [6]. Consequently, process disturbance following a temperature increase may be caused by the higher ammonia level, even though the nitrogen load caused no disturbance at the lower temperature. Accordingly, the higher ammonia concentration in D42 and D^TE^42 compared with D37 and D^TE^37 (Table 2) could be a possible contributor to the differences observed between these digesters.

Induced accumulation of propionate seems to be a frequent concern during operation within the higher mesophilic temperature range [28, 45, 46]. In accordance higher propionate levels were throughout the course of the present study detected in D42 compared to D37. The propionate to acetate ratio has been cited as an early indicator of imminent process failure [47], which indicates that the 42 °C digester would be less resistant to the appearance of stress or increased loading than D37. The reason for the elevated propionate level throughout in D42 and in the initial period in digester D^TE^42 was not fully established, but could be due to sub-optimal temperature conditions for growth of SPOB (reviewed in [48]) or increased inhibition of the active propionate-degrading community by the increasing ammonia level. However, the decrease in propionate level after day 120 in D^TE^42 rather implies slow microbial acclimatisation to the prevailing operating conditions, which was obviously possible when there was adequate access to trace elements.

Moreover, the disappearance of accumulated propionate, the increased methane yield and the differences in the kinetic profile in D^TE^42 after the extended operating period at constant parameters (including the initial acclimatisation period of 3 months) emphasise the importance of having an extended operating period in experimental trials before claiming stable digester performance.

#### Abundance and composition of methanogens, acetogens and SAOB

The methanogenic and acetogenic community structure and the abundance of SAOB and methanogens were strongly influenced by the operating temperature. The higher level of *Methanomicrobiales* and *Methanosarcinaceae* observed with increased operating temperature is in line with previous observations [28]. However, the impact of ammonia, which was present at considerably higher levels in the 42 °C digesters, needs to be considered in this context. For example, the high abundance of OTU20 in the *mcrA*-gene profile and T-RF 116 bp (OTU_*fhs*_28) in the putative acetogenic community profile not only indicates favourable temperature conditions for the populations present, but also their considerably high ammonia tolerance. Constrained competition for substrate induced by the higher ammonia level could have been another advantage for these genotypes. Similarly, the lower competitiveness from other more ammonia-sensitive acetate consumers in the 42 °C digesters probably promoted increased abundance of the ammonia-tolerant SAOB. However, the higher growth rate at 42 °C than at 37 °C of known syntrophic acetate-degrading cultures [26] implies that the populations directly benefited from the increased temperature. In a previous study, a temperature rise increased the abundance of *T. acetatoxydans* in a high-ammonia (0.6 g NH_3_/L) digester, but *S. schinkii* and *C. ultunense* were not affected [28].

### Functional gene richness, evenness and temporal dynamics

The *mcrA*-gene profiles indicated considerably higher methanogenic richness and evenness (here defined as the number and relative abundance of the identified OTUs) in digesters operating at 37 °C than at 42 °C. The impact of temperature on potential acetogenic community richness (based on number and relative abundance of T-RFs) was not completely explained by the T-RFLP profiles, since D^TE^37 had higher numbers than D42 and D^TE^42. Nevertheless, the *fhs* clone libraries retrieved from D^TE^37 and D^TE^42 clearly displayed higher richness at the lower temperature than at the higher temperature with TE addition. The uncut portion of sequences at the lower temperature constituted a threefold higher number of different amplified partial *fhs* genotypes than at the higher temperature. Still, despite low methanogenic temporal dynamics and the considerably low species richness and evenness in acetogenic and methanogenic communities in the 42 °C digesters, functional stable operation was possible, in particular with TE addition. However, the microbial analyses strongly indicated low robustness to disturbances of these digesters.

Interestingly, at 42 °C TE addition did not seem to substantially affect the methanogenic and acetogenic functional gene richness and evenness, since a few prominent populations characterised the communities of both D^TE^42 and D42 (Figs. 3, Additional file 1: Figures S2, S3). On the other hand, at 37 °C TE addition promoted higher richness and evenness within the methanogenic community. In particular, higher strain richness of *M. bourgensis* was apparent in D^TE^37 than in D37. This led us to speculate that the comparatively high richness and evenness in D^TE^37 could indicate a possible link between the microbial community structure and the enhanced performance of this digester.

Mesophilic conditions (32–37 °C) have previously been reported to promote higher microbial diversity than thermophilic conditions (52–55 °C) [49, 50]. The results of the present study imply that a temperature increase of only 5 °C severely restricts the richness of functional gene composition of both the methanogenic and potential acetogenic communities. The strong effect of temperature observed here could also have been augmented by the temperature-induced enhancement of ammonia. The importance of high community diversity, commonly regarding species richness and evenness, for functional stability and robustness to stress has frequently been reported [51–55]. However, high functional stability in the degradation of complex substrates has been shown to be achievable despite low bacterial diversity [56], and community flexibility has instead been suggested as a vital factor for process performance [57]. Werner et al. [58] postulated that temporal population changes allow for rapid adjustment of the community following perturbations and disturbances, thereby contributing to a more robust process. In the present study, gene copy numbers, as estimated by quantitative PCR, revealed temporal changes in *Methanomicrobiales* in D37 and D^TE^37, whereas in D42 and D^TE^42 the abundance of this methanogenic order was relatively constant throughout the trial. The profiling of the acetogenic community at 37 °C also indicated alterations in the composition over time, whereas no such indications were obvious at the higher temperature.

### Syntrophic propionate oxidation

The accumulation of propionate, together with high acetate concentration, is considered to be the major problem in digesters with high ammonia concentrations and trace element deficiency, so the propionate degradation pathway is of great interest. In digesters with limited levels of sulphate, syntrophic degradation coupled with hydrogen removal is believed to be the major pathway for propionate degradation. Syntrophic propionate communities have been studied in anaerobic digesters [59, 60]. However, to our knowledge the impact of ammonia on the syntrophic propionate community has not been investigated to date. Unfortunately, the molecular methods applied here did not reveal great insights into this intriguing community. For further analysis of the SPOB populations in high-ammonia digesters, primer optimisation and enrichment cultivation are essential. However, such work was beyond the scope of the present study.

## Conclusions

Temperature alteration and trace element addition are strategies known to effect digester performance and microbial communities under high ammonia stress. In the present study, involving a parallel, long-term operation of digesters at high ammonia and comprehensive analyses of syntrophic acetate-oxidising populations and the acetogenic and methanogenic communities, we have been able to link significant microbial community dynamics with these operating changes and relate them to the enhanced performance.

Interestingly, the influence of TE supplementation strongly depended on temperature. At 37 °C addition of TE considerably enhanced the digester performance, rebutting that SAO-dominant processes inevitably involve operation at high VFA levels and low methane yield. The microbial community in this well-performing digester was distinguished by high abundance and high population richness of *M. bourgensis*, emphasising the importance of this methanogenic group for successful operation in high-ammonia, mesophilic conditions. A putative acetogenic population that significantly increased in abundance by TE addition at 37 °C was detected, indicating an important role in the well-performing process, whereas the known SAOB *S. schinkii* decreased in abundance. At 42 °C the effect of TE addition on the community structure was substantially less distinct, most likely due to co-occurrence of loss of species richness. Even though the performance of D^TE^42 gradually enhanced throughout the course of the study the temporal dynamics within methanogenic and acetogenic community structures were relatively small. However, also at this temperature the TE addition decreased the abundance of known SAOB (*S. schinkii* and *T. acetatoxydans*).

Another notable finding in this study was that just a slight temperature increase of 5 °C had major influence on microbial community structure, including considerably decreased richness and evenness of methanogenic and acetogenic communities. The acetogenic community was even characterised by a dominance of a single genotype. This temperature alteration furthermore increased the abundance of *Methanomicrobiales*, *Methanosarcinaceae* and known SAOB, in line with our initial hypothesis.

In overall, the recovery of the *fhs* genes in digesters dominated by SAO confirms the importance of the species represented for anaerobic degradation in high-ammonia conditions and supports their position as potentially acetate-oxidising syntrophs. Genotypes identified to be significantly affected by TE addition and/or temperature indicate a possible link to altered digester performances and are important candidates for further research. Noteworthy of this study was that functional stable operation was possible at the higher temperature despite the low temporal dynamics, evenness and species richness of acetogenic and methanogenic communities. The findings of this study are valuable in the development of interventions for improved process operation.

## Methods

### Anaerobic digester operation

Four identical laboratory-scale continuously stirred tank digesters (Belach Bioteknik, Stockholm, Sweden) with active volume of 5 L were operated in parallel for 320 days. The inoculum used for start-up was taken from a high-ammonia (5.5 g NH_4_^+^-N/L) mesophilic digester characterised by dominance of SAO [16] and high abundance of SAOB and hydrogenotrophic methanogens [35]. Material from this high-ammonia digester was withdrawn after more than 440 days of operation and stored for several years at 37 °C, occasionally with addition of feedstock, before being used as inoculum for the digesters in this study.

Semi-continuous operation was achieved by daily batch feeding 6 days a week with source-sorted organic fraction of municipal solid waste supplemented with egg albumin powder, i.e. the same substrate as used in the original digester from which inoculum was taken [16]. The OLR was set to 2.3 g VS/(L day) and the hydraulic retention time (HRT) to 30 days. The reference digester (D37) operated at 37 °C and digester D42 at a temperature of 42 °C. The other two digesters received a supplementary mixture (in this report referred to as TE; BDP-868, Kemira Oyj, Helsingborg, Sweden) containing iron (Fe^2+^/Fe^3+^), cobalt, nickel, selenium, tungsten and hydrochloric acid and operated at 37 °C (digester D^TE^37) or 42 °C (digester D^TE^42). The dosage of the TE mixture to D^TE^37 and D^TE^42 was 0.009 L/kg digester sludge. The digesters were allowed to adjust to the prevailing operating conditions for 3 months before the start of performance monitoring and molecular analyses. The starting point for these analyses is henceforth referred to as operating day 0.

### Digester monitoring

Measurement of total gas production, biogas composition, pH, total solids (TS) and VS was performed as described previously [61]. For calculating degree of degradation, the rough estimation method recommended elsewhere [62] was applied in order to compensate for VFA losses in the determination of dry matter. Methane content in the gas and VFA concentrations (acetate, propionate, butyrate, isobutyrate, valerate, isovalerate, capronate and isocapronate) were measured using gas chromatography (GC) and high performance liquid chromatography (HPLC), respectively [61]. All volumetric gas values presented here are converted to standard conditions at pressure 1.01325 bar and temperature 273.2 K. Ammonium-nitrogen was determined in period 2 according to standard methods [63]; the ammonia concentration was calculated from the equilibrium relationship described elsewhere [10]. Sulphide concentration was measured with a Biogas 5000 Analyser (Geotechnical Instruments). Metal analyses in digester samples, taken in periods 1 and 3, were performed at the Dept. of Thematic Studies (Linköping University, Sweden) according to Swedish Standard SS028311. Total metal content was extracted by digestion of triplicate sludge samples with 7 M nitric acid in an autoclave at 120 °C for 30 min using inductively coupled plasma mass spectrometry (ICP-MS, NexION 300, Perkin Elmer, USA). Blanks and reference material (CRM 029050, RTC, recovery of >90 % of the certified values) were analysed for quality control.

### Methanogenic pathway

The pathway for conversion of acetate to methane was determined by tracer analysis, involving incubation of digester sludge (20 mL) with [2-^14^C]-acetate (final concentration 0.11 µCi/mL) and monitoring of labelled gases by scintillation counting as described by Schnürer and Nordberg [16]. A ^14^CO_2_/^14^CH_4_ ratio above 1 indicates dominance of SAO, while aceticlastic methanogenesis is the main pathway at ratios below 1 [16].

### Kinetics study

Hydrogen partial pressure was analysed by GMH 3111 (Greisinger Electronic, GmbH) and samples for analyses were taken before feeding and repeatedly for several hours after feeding. By determining total gas production and methane content at fixed intervals after feeding, the kinetics of methane production in the anaerobic digesters were estimated after 20, 160 and 311 days of operation, in order to represent operating period 1, 2 and 3, respectively. The relative hydrogen partial pressure in the digesters was analysed on days 160 and 311.

### Batch degradation assays

Batch assays were conducted on biomass taken from the different digesters after 150 days of operation, i.e. in period 2. Triplicate cultures from each digester were prepared in 250-mL serum bottles containing 7.5 gVS/L digestate, 3 gVS/L substrate and water to reach a total volume of 193 mL. The substrate was identical to that of the parent digester, i.e. municipal waste supplemented with egg albumin, with (D^TE^37, D^TE^42) or without (D37, D42) TE addition. Three replicate cultures without substrate were operated as controls for each set of assay conditions. Incubation was performed at the respective process temperature (37 or 42 °C) in a shaking incubator (90 rpm, Grant OLS200). Gas and liquid samples were taken continuously and analysed for methane content and VFA concentration by GC and HLPC as described above. Net methane yield and specific VFA concentration were calculated by subtracting the average value obtained for the corresponding control assays from the results of each batch assay.

### Molecular analyses

Samples for molecular analyses were withdrawn from the digesters after 49, 77, 126, 168, 253 and 280 days of operation and stored at −20 °C until analysis. Extraction of total genomic DNA with the FastDNA Spin kit for soil (Qbiogene, Illkrich, France) was performed as recommended by the supplier. To circumvent eventual inhibition due to requirement of high DNA content in the PCR for the syntrophic propionate-oxidising community analyses, the procedure for extraction of DNA was complemented with an extra purification step. The sample was then washed and resuspended with 5.5 M guanidine thiocyanate before transfer to SPIN filter, following settling of the binding matrix. Triplicate DNA extractions were prepared from each sampling point. Genomic DNA of pure archaeal and bacterial cultures was recovered using the DNeasy Blood and Tissue kit (Qiagen).

#### Quantitative PCR analyses

Construction of DNA standards and qPCR analyses performed on syntrophic acetate oxidisers (*C. ultunense*, *S. schinkii* and *T. acetatoxydans*) and the methanogenic population (*Methanomicrobiales*, *M*. *bourgensis*, *Methanosarcinaceae*, *Methanosaetaceae* and *Methanobacteriales*) as described previously [20, 35]. Total bacteria were assayed as reported elsewhere [64]. Triplicate samples from each digester and sampling point were included in the analyses. The mean similarities of gene abundances were compared using Student’s *t* test and *P* < 0.05 was regarded as statistically significant. In order to monitor artefacts such as primer dimer formation and to assess whether non-specific amplification had occurred, a temperature melt curve was performed at the end of each qPCR assay (55–95 °C, Δ*T* = 0.1 °C/s). Non-specific amplification was further evaluated by analysing the products formed, using gel electrophoresis. All standard curves for the quantitative PCR analyses had a linear correlation coefficient (*r*^2^) of 0.98–1.0 and the calculated qPCR efficiency of the reactions varied between 80 and 100 %. The detection limit for the different groups was about 60–560 gene copy numbers/mL digester sludge.

Presence of the thermophilic SAOB *T. phaeum* and *T. lettingae* was analysed with end-point PCR as described by Sun et al. [19]. Due to generation of unspecific amplification by primer pair Mst in the absence of strains related to *Methanosaetaceae*, the products formed in the qPCR analyses were checked by performance of clone library as described by Sun et al. [19].

#### Terminal restriction fragment length polymorphism and statistical analysis

The acetogenic community was profiled with DNA samples extracted on days 77, 168 and 280, in order to represent the three operating periods of the digesters. T-RFLP was performed using primers targeting the functional gene encoding the enzyme FTHFS. The recently designed degenerated 3-SAO*fhs* primer pair, optimised to include SAOB, was used in PCR as described previously [22]. The 3-SAO*fhs* forward primer was labelled with 6-carboxyfluorescein (FAM). Each preparation was then subjected to triplicate PCR reactions and the corresponding PCR products were pooled to reduce potential bias. To reduce background noise, the respective band at approx. 635 bp was gel-purified using the QIAquick Gel Extraction Kit (Qiagen, Hilden, Germany). The PCR fragment was digested with *Hpy*188III (Fermentas) at 37 °C for 3 h and T-RFs were separated by ABI3730XL DNA analyser (Applied Biosystems) at Uppsala Genome Center (Sweden), using MapMarker 1000 (Rox) as size standard. The fragment data obtained were evaluated with Peak Scanner software (Applied Biosystems) including peaks ranging from 50 to 635 bp. The resulting profiles were further evaluated in Excel by setting the threshold value for peak abundance at 1 % of total peak abundance.

In order to formally assess which T-RFs were influenced by TE, temperature or a combination of these, the following statistical analysis was performed. Four environmental settings were considered: core (D37), addition of TE (D^TE^37), temperature (D42) and TE addition combined with temperature (D^TE^42). For each of these settings, the frequency of each T-RF was observed at three different time points, days 77, 168 and 280. Each T-RF in the core was assessed for association with TE or temperature by comparing frequency at all sampling points using a paired *t* test. This pairing eliminated the effect of time in the dataset. The *t* test was actively chosen instead of a non-parametric test, because it evaluated the actual numerical frequency levels rather than just their ranks, which was deemed more relevant to the data. *P* values <0.05 indicated TE or temperature influence. The combined effect of TE and temperature was assessed by a *t* test investigating whether the frequency was similar to the expected effect of TE (as estimated with the TE *t* test) plus the expected effect of temperature (as estimated with the temperature *t* test). A low interaction (*P* < 0.05) indicated that the effect of the combined treatment was significantly different from that of the individual treatments.

#### Construction of clone libraries and phylogenetic trees

To further investigate the microbial communities, clone libraries from digesters D^TE^37 and D^TE^42 (day 168) for the acetogenic communities and all four digesters (day 280) for the methanogenic communities, were constructed. Triplicate DNA extractions were used as template in PCR amplification with the 3-SAO*fhs* primer pair [22], or with primers mlas and mcrA-rev using recommended reaction conditions [65]. In both surveys, amplicons of correct size from the triplicate DNA extractions were pooled, gel-purified and subsequently cloned into the pGEMT vector system (Promega, Madison, WI, USA) as recommended by the manufacturer. Cells of CaCl_2_-competent *Escherichia coli* JM109 (Promega) were transformed by the ligation mixture according to the manufacturer`s protocol. Clones were randomly selected and plasmid inserts were verified with colony PCR using the vector-specific primer pair M13. The amplicons were subsequently sequenced with the M13 primer (Macrogen Inc. Seoul, Korea). Quality checks, editing and sequence assembly were performed with Geneious v6.1 [66]. FTHFS homologues with nucleotide identity above 97 % were considered to represent the same genotype and assigned to an OTU_*fhs*_. In order to reveal differences within the archaeal community on strain level, the *mcrA* gene sequences were assigned to an OTU based on 99 % nucleotide identity. Phylogenetic analyses were conducted with the BlastP search algorithm (National Library of Medicine) using a representative sequence from each OTU. Transformation of the sequences into amino acid sequences, followed by homology search ≥89 % amino acid sequence similarity, identified affiliation to methanogenic species [65, 67].

The *mcrA* sequences were aligned by MUSCEL v5.2.2 [67] and a maximum likelihood tree was constructed using PhyML v3.0 [68] choosing the WAD substitution matric. Both MUSCEL v3.8.31 and PhyML v3.0 are available on the Mobyle platform of Institute Pasteur. FTHFS multiple sequence alignment was performed using MAFFT v7.017 [69] and the maximum likelihood tree was constructed using PhyML v3.0, both implemented in Geneious v6.1.8 (Biomatters Ltd., USA). The tree includes partial *fhs* sequences obtained in this study, obtained by [33] and [70], selected public available partial *fhs* sequences (Genbank Popsets JQ082213-54; JQ082255-97; submitted by B. Müller and A. Schnürer) and reference strains (accession numbers given in brackets).

#### Molecular assessment of syntrophic propionate-oxidising bacteria and establishment of mcrA genes of methanogenic isolates

End-point PCR with the general pct primers and the specific primers pct-c1 to -c7 designed to target the propionate CoA-transferase gene (*pct*) [60] were used for molecular assessment of syntrophic propionate oxidisers in the digesters. Genomic DNA of *P. propionicicum* (DSM 15578), *S. fumaroxidans* (DSM 10017), *S. pfennigii* (DSM 10092), *S.wolinii* (DSM 2805M), *S.sulfatireducens* (DSM 16706) and *T. acetatoxydans* (isolated at the Dept. of Microbiology, Swedish University of Agricultural Sciences) was subjected to PCR with the pct primers to check possible product formation. The PCR procedure was initially performed as described elsewhere [60]. However, due to low concentration of products formed on applying the recommended conditions with pure culture DNA, changes in the PCR reaction and in the protocol were tested in order to improve the outcome. Alterations in concentrations of DNA, primers, BSA or MgCl_2_ were assessed, and different annealing temperatures and a touchdown programme (95 °C for 5 min, 10 cycles of 30 s at 95 °C, 45 s at 62 °C (decreased by 1 °C per cycle to 53 °C), and 72 °C for 90 s, followed by 30 cycles of 30 s at 95 °C, 45 s at 53 °C, and 72 °C for 90 s) were evaluated. PCR products were verified by agarose gel electrophoresis, and expected bands (~1300 bp) were gel-purified and sequenced as described above. In order to include the strains using the alternative dismutation pathway for syntrophic propionate oxidation [71], primers targeting *Smithella*-related species (Smi103-f: 5′-CGCGTGGATAATCTACCCCTC-3′; Smi442-r: 5′-GCTCGACAGAGCTTTACGGT-3′) were designed. 16S rRNA sequences of known syntrophs and closely related species were aligned in Geneious v6.1 with ClustalW, group- or species-specific primers were designed and the specificity was checked by Blast. The 16S rRNA specific primer pair Synt737-f (5′-CTGGAGAGGAAGGGGGAATT-3′) and Synt906-r (5′-ATGAGTACCCGCTACACCT-3′) was also designed to specifically study eventual presence of *S. wolinii*, *S. pfennigii*, *S. fumaroxidans* and *S. sulfatireducens*. In targeting the 16S rRNA gene, the PCR protocol consisted of initial denaturation at 94 °C for 5 min, 40 cycles of denaturation at 94 °C for 30 s, annealing at 61 °C for 40 s, and elongation at 72 °C for 30 s, followed by a final extension step of 4 min at 72 °C.

The *mcrA* genes of the isolates *M.bourgensis* sp. BA1, MAB1, MAB2 and MAB3 [17] were obtained using the forward primer ME1 [72] or primer mlas together with the reverse primer mcrA-rev. The amplification was performed with genomic DNA extractions from pure cultures, using the PCR manual described elsewhere [65].

#### Nucleotide sequence accession numbers

All sequences were deposited in the NCBI GenBank database under the following accession numbers: GenBank: KJ701041-KJ701114 for all *fhs* sequences and GenBank: KJ701115-KJ701185 for all *mcrA* sequences. The *mcrA* sequence data for *M. bourgensis* strains BA1, MAB1, MAB2 and MAB3 are available at the accession numbers GenBank: KJ708787-KJ708790.

## Additional file

Additional file 1: Table S1. Total metal concentrations in digester samples.** Table S2.** Accession numbers of* mcrA* sequences and closest relative based on nucleotide sequence.** Table S3.** Accession numbers of* fhs* sequences retrieved in clone libraries.** Figure S1.** Acetate and propionate degradation profiles in a batch assay experiment.** Figure S2.** Acetogenic community composition according to T-RFLP of* fhs* gene amplicons.** Figure S3.** Relative distributions of partial* fhs* genes retrieved in construction of clone libraries.

## Abbreviations

TE: trace element mixture including iron (Fe^2+^/Fe^3+^), cobalt, nickel, selenium, tungsten and hydrochloric acid
SAOB: syntrophic acetate-oxidising bacteria
VFA: volatile fatty acid
CODH: carbon monoxide dehydrogenase
tAD: thermophilic anaerobic digestion
SAO: syntrophic acetate oxidation
HM: hydrogenotrophic methanogenesis
qPCR: quantitative PCR
*mcrA*: methyl coenzyme-M reductase
OTU: operational taxonomic unit
FTHFS: formyltetrahydrofolate synthetase
T-RFLP: terminal restriction fragment length polymorphism
T-RF: terminal restriction fragment
SPOB: syntrophic propionate-oxidising bacteria
OLR: organic loading rate
HRT: hydraulic retention time
TS: total solids
VS: volatile solids
HPLC: high performance liquid chromatography
*pct*: propionate CoA-transferase gene
BSA: bovine serum albumin
