# Supplementary material for: Trace element and temperature effects on microbial communities and links to biogas digester performance at high ammonia levels
Source: Biotechnol Biofuels. 2015 Sep 22;8:154. doi: 10.1186/s13068-015-0328-6 (PMC4578335; doi:10.1186/s13068-015-0328-6)
Supplement: Supplementary file 1 — Additional file 1: Table S1. Total metal concentrations in digester samples. Table S2. Accession numbers of mcrA sequences and closest relative based on nucleotide sequence. Table S3. Accession numbers of fhs sequences retrieved in clone libraries. Figure S1. Acetate and propionate degradation profiles in a batch assay experiment. Figure S2. Acetogenic community composition according to T-RFLP of fhs gene amplicons. Figure S3. Relative distributions of partial fhs genes retrieved in construction of clone libraries. [file 13068_2015_328_MOESM1_ESM.docx]

**Additional files**

**Table S1 Total metal concentrations in digester samples taken in periods 1 and 3.** Se and Mo were below the detection limit in all samples taken in period 3

| Element | **Fe [mg/kg TS]** | | | **Co [mg/kg TS]** | | **Ni [mg/kg TS]** | | **Se**  **[mg/kg TS]** | **Mo**  **[mg/kg TS]** |
| --- | --- | --- | --- | --- | --- | --- | --- | --- | --- |
| Operating period | **1** | | **3** | **1** | **3** | **1** | **3** | **1** | **1** |
| **D37** | 4000±100 | | 5800±700 | <3 | 4±0 | 11±1 | 18±1 | <2 | <2 |
| **D^TE^37** | 20000±1200 | 19000±900 | | 20±1 | 12±1 | 212±9 | 25±1 | <2 | 45±3 |
| **D42** | 5100±200 | 5600±200 | | <3 | 4±0 | 13±0 | 15±1 | <2 | <2 |
| **D^TE^42** | 23000±800 | 20000±800 | | 20±0 | 12±1 | 41±1 | 26±1 | <2 | 8±1 |

**Table S2 Accession numbers of *mcrA* sequences and closest relative based on nucleotide sequence.** The clones were retrieved from the digesters on day 280. In total, 62, 89, 58 and 87 clones from digester D37, D^TE^37, D42 and D^TE^42, respectively, were sequenced and analysed

| **Accession number(s)** | **Assignment** | **Digester (number of clones)** | **Closest relative^1^ (nucleotide identity)** |
| --- | --- | --- | --- |
|  |  |  |  |
| KJ701124-27 | OTU1 | D37 (26) | <79% |
| KJ701128-32 | OTU2 | D^TE^37 (17) | *Methanoculleus bourgensis* sp. MS2 (92%) |
| KJ701133-37 | OTU3 | D37 (5); D^TE^37 (6) | *M. bourgensis* sp. MS2 (99%) |
| KJ701139-42 | OTU4 | D37 (6); D^TE^37 (5) | *M. bourgensis* sp. MS2 (99%) |
| KJ701143-48 | OTU5 | D37 (8); D^TE^37 (5) | *M. bourgensis* sp. MS2 (99%) |
| KJ701149-53 | OTU6 | D37 (6); D^TE^37 (4) | *M. bourgensis* sp. MS2 (99%) |
| KJ701154-57 | OTU7 | D^TE^37 (7) | *M. bourgensis* sp. CB1 (99%) |
| KJ701158-61 | OTU8 | D37 (1); D^TE^37 (7) | *M. bourgensis* sp. MS2 (99%) |
| KJ701162-64 | OTU9 | D^TE^37 (6) | *M. bourgensis* sp. MS2 (99%) |
| KJ701165-69 | OTU10 | D37 (3); D^TE^37 (2) | *M. bourgensis* sp. MS2 (99%) |
| KJ701170-71 | OTU11 | D^TE^37 (5) | *M. bourgensis* sp. MS2 (99%) |
| KJ701172-73 | OTU12 | D^TE^37 (2) | *M. bourgensis* sp. MS2 (97%) |
| KJ701174 | OTU14 | D^TE^37 (4) | *Methanomassiliicoccus luminyensis* (83%) |
| KJ701175 | OTU15 | D^TE^37 (4) | *M. bourgensis* sp. MS2 (92%) |
| KJ701176-77 | OTU17 | D37 (3) | *Methanobrevibacter smithii* (89%) |
| KJ701178-79 | OTU19 | D37 (2); D42 (5); D^TE^42 (4) | <79% |
| KJ701180-84 | OTU20 | D37 (2); D42 (53); D^TE^42 (81) | *M. bourgensis* sp. MS2 (93%) |
| KJ701185 | D^TE^42_cl27 | D^TE^42 (2) | <79% |
| KJ701115 | D^TE^37_cl6 | D^TE^37 (1) | *M. bourgensis* sp. CB1 (97%) |
| KJ701116 | D^TE^37_cl19 | D^TE^37 (1) | *M. bourgensis* sp. MS2 (96%) |
| KJ701117 | D^TE^37_cl26 | D^TE^37 (1) | *M. bourgensis* sp. MS2 (96%) |
| KJ701119 | D^TE^37_cl29 | D^TE^37 (1) | *M. bourgensis* sp. MS2 (94%) |
| KJ701120 | D^TE^37_cl36 | D^TE^37 (1) | *M. bourgensis* sp. MS2 (95%) |
| KJ701123 | D^TE^37_cl52 | D^TE^37 (1) | *M. bourgensis* sp. MS2 (94%) |
| KJ701118 | D^TE^37_cl26_2 | D^TE^37 (1) | *M. bourgensis* sp. MS2 (94%) |
| KJ701121 | D^TE^37_cl36_2 | D^TE^37 (1) | *M. bourgensis* sp. CB1 (97%) |
| KJ701122 | D^TE^37_cl39_2 | D^TE^37 (1) | *M. bourgensis* sp. MS2 (96%) |

^1^<79% = less than 79% identity to available *mcrA* genes of previously characterised species.

**Table S3 Accession numbers of *fhs* sequences retrieved in clone libraries from digesters D^TE^37 and D^TE^42 on day 168.** All *fhs* sequences had low nucleotide sequence identity (<79%) to previously characterised species

| **Accession number(s)** | **Assignment in T-RFLP^1^, phylogenetic tree**  **and in clone library** | **Digester (number of clones)** | **Close relateness (≥99%) to**  **previous sequences**  **(accession number)** |
| --- | --- | --- | --- |
|  |  |  |  |
| KJ701082-84 | 635b [OTU*_fhs_*21] | D^TE^37 (6) | <79% |
| KJ701061-64 | 496bp [OTU*_fhs_*22] | D^TE^37 (6) | **-** |
| KJ701041-45 | 65bp [OTU*_fhs_*23] | D^TE^37 (6) | - |
| KJ701059/60 | 455bp [OTU*_fhs_*24] | D^TE^37 (3) | - |
| KJ701086/87 | 635bp [OTU*_fhs_*25] | D^TE^37 (4) | <79% |
| KJ701088/89 | 444bp [OTU*_fhs_*26] | D^TE^37 (1); D^TE^42 (1) | <79% |
| KJ701066-68 | 581bp [OTU*_fhs_*27] | D^TE^37 (3) | - |
| KJ701046-57 | 116bp [OTU*_fhs_*28] | D^TE^42 (39) | - |
| KJ701058 | 309bp | D^TE^37 (2); D^TE^42 (1) | JQ082286, HM365339 |
| KJ701074 | 90bp | D^TE^37 (1) | JQ082258 |
| KJ701101 | 283bp [OTU*_fhs_*29] | D^TE^42 (3); D^TE^37 (2) | JQ082274 |
| KJ701104 | 378bp [OTU*_fhs_*30] | D^TE^42 (3); D^TE^37 (3) | JQ082296 |
| KJ701093 | 635bp [OTU*_fhs_*31] | D^TE^42 (7) | JQ082263 |
| KJ701096 | 635bp [OTU*_fhs_*32] | D^TE^42 (1); D^TE^37 (5) | JQ082293 |
| KJ701110 | 635bp [OTU*_fhs_*33] | D^TE^37 (14) | JQ082213 |
| KJ701065 | 522bp | D^TE^37 (2) | - |
| KJ701069 | 378bp | D^TE^37 (1) | - |
| KJ701070 | 80bp | D^TE^37 (1) | - |
| KJ701071 | 240bp | D^TE^37 (1) | - |
| KJ701072 | 83bp | D^TE^37 (1) | - |
| KJ701073 | 90bp | D^TE^37 (1) | - |
| KJ701075 | 469bp | D^TE^37 (1) | - |
| KJ701076 | 635bp | D^TE^42 (1) | - |
| KJ701077 | 581bp | D^TE^42 (1) | - |
| KJ701078 | 588bp | D^TE^37 (1) | - |
| KJ701079 | 90bp | D^TE^42 (1) | - |
| KJ701080 | 309bp | D^TE^42 (1) | - |
| KJ701081 | 635bp | D^TE^37 (2) | <79% |
| KJ701085 | 635bp | D^TE^37 (4) | - |

^1^If assigned as 635bp in T-RFLP, the corresponding sequence indicated no restriction site for *Hpy*188III.

**Figure S1 Acetate and propionate degradation profiles in a batch assay experiment.** The batch assays were conducted with biomass taken from the different digesters after 150 days of operation (period 2). The substrate was identical to that in the parent digester, i.e. municipal waste supplemented with egg albumin, with (D^TE^37, D^TE^42) or without (D37, D42) addition of trace elements (^TE^). In batch assays with R37 and D47 as parental digesters, isovalerate appeared at net concentrations of up to 0.6-0.8 g/L (not shown).


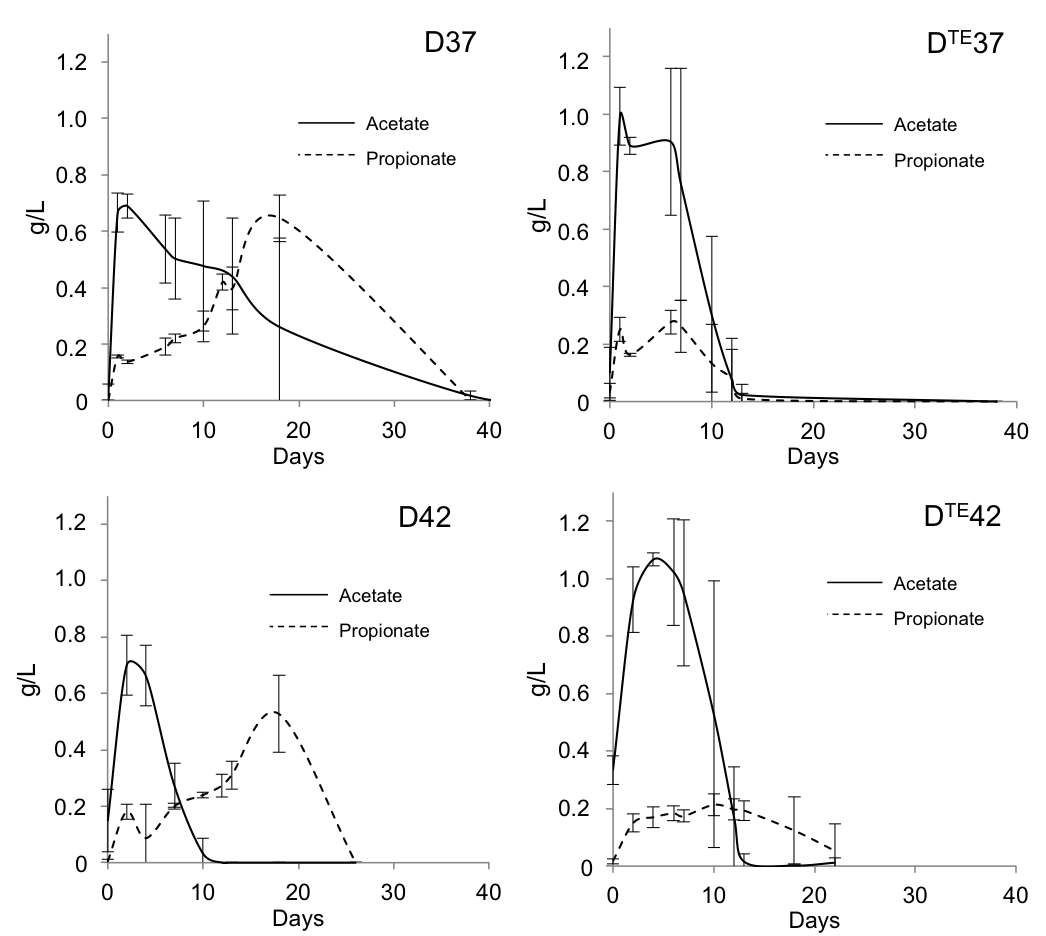


**Figure S2 Acetogenic community composition according to T-RFLP of *fhs* gene amplicons.** The T-RFLP analyses were assessed on samples from digesters D37, D^TE^37, D42 and D^TE^42 on day 77, 168 and 280, representing period 1, 2 and 3, respectively. Accession numbers of partial sequences of cloned *fhs*-genes affiliated to the T-RFs are specified in Table S3 and Figure 6. The 86 bp T-RF was not assigned to any gene recovered from the D^TE^37 clone library. Only T-RFs comprising at least 1% relative abundance are shown in the graph.

T-RF 55 T-RF 65 T-RF 86 T-RF 116 T-RF 283 T-RF 455

T-RF 581 T-RF 635


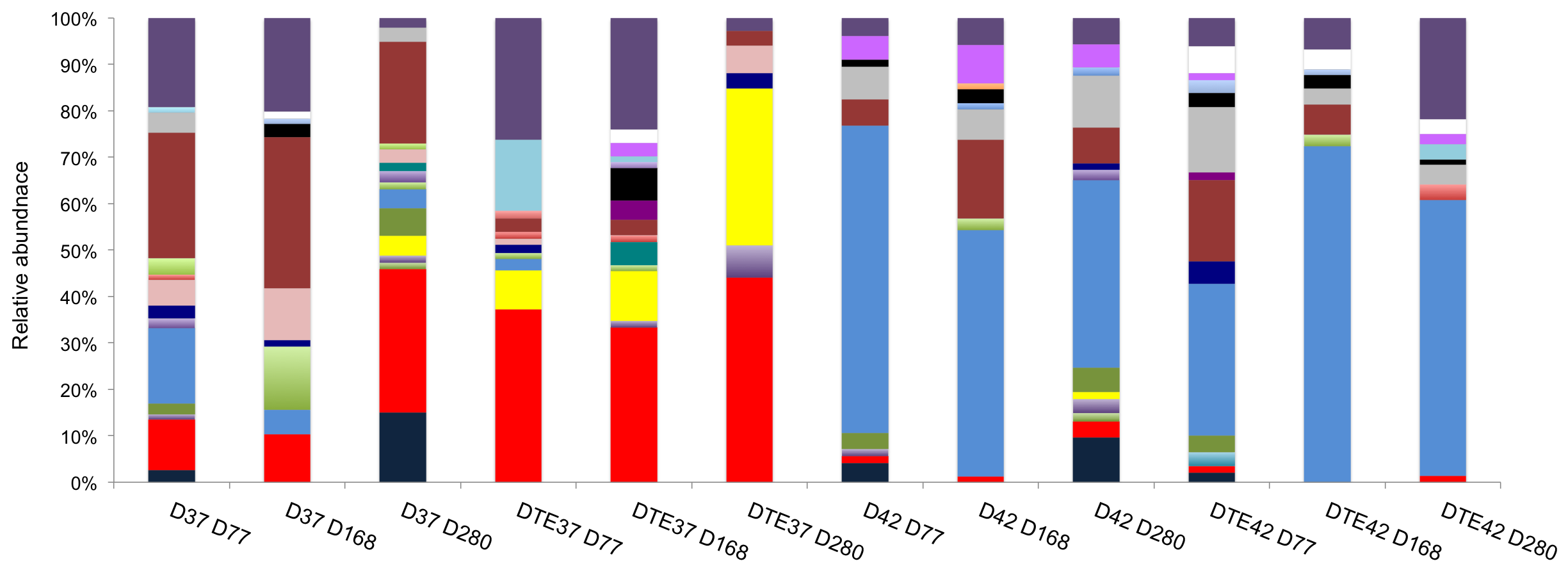


**Figure S3 Relative distributions of partial *fhs* genes retrieved in construction of clone libraries from digesters D^TE^37 and D^TE^42 on day 168 (period 2).** The size (bp) of the T-RFs is given in brackets. Frequencies and identities of the OTUs are listed in Table S3.
